# Attitudes of patients with psychotic disorders towards psychoactive substances

**DOI:** 10.1192/j.eurpsy.2025.2274

**Published:** 2025-08-26

**Authors:** P. Tokić, T. Mastelić, N. Rančić, D. Vukorepa, A. Čikara, J. Erceg, A. Rađa, T. Borovina Marasović, S. Kozina, D. Lasić, T. Glavina

**Affiliations:** 1 Department of Mental Health, Split-Dalmatia County Teaching Institute of Public Health; 2 Department of Psychiatry, University Hospital of Split; 3 Department for Psychological Medicine; 4 Department of Psychiatry, School of Medicine University of Split, Split, Croatia

## Abstract

**Introduction:**

Understanding patient attitudes toward psychoactive substances is essential for improving treatment outcomes in psychotic disorders. Substance use complicates these disorders, worsening symptoms and hindering recovery. Despite known risks, many individuals with psychotic disorders engage in substance use influenced by various factors. The debate over the legalization, decriminalization, and normalization of psychoactive substances, including marijuana and psychedelics, is growing. Legalization removes legal restrictions, decriminalization reduces penalties, and normalization involves societal acceptance of these substances. Alcohol, a culturally embedded substance, is also highly harmful despite its legal status. Recently, there has been increased interest in cannabis for therapeutic use, though its role in psychotic disorders remains contentious. While some evidence suggests the benefits of cannabidiol (CBD), excessive use of high-THC cannabis may elevate the risk of psychosis or exacerbate symptoms.

**Objectives:**

To examine the attitudes and perceptions of patients with psychotic disorders toward alcohol, marijuana, and psychedelics, and to assess the prevalence of psychoactive substance use among individuals with psychotic disorders.

**Methods:**

For this descriptive study, data were collected from September 2023 to September 2024 through a questionnaire distributed to patients during their hospitalization at the University Hospital of Split in the Psychiatry Department. Inclusion criteria included adult patients with ICD diagnoses from F20-F29 who agreed to participate in the study; exclusion criteria included patients with F21 (unless they also have F23) and those who declined to participate.

**Results:**

This study examined the attitudes and behaviors of 62 patients with psychotic disorders regarding alcohol and psychoactive substance use. The sample consisted of 37 men (59.7%) and 25 women (40.3%). Alcohol consumption was reported by 34 respondents (54%) in socially acceptable quantities, while 7 respondents (11.3%) admitted to combining alcohol with medications. Additionally, 8 respondents (12.9%) reported using other psychoactive drugs. Regarding perceptions, 6 respondents (9.7%) believed that marijuana helps their health, and an equal number expressed a similar belief about psychedelics.

**Conclusions:**

Despite therapeutic cooperation, many patients continue to consume alcohol due to its availability and social acceptance. Some patients use marijuana, believing that it improves their mental state, while others use psychedelics; however, fewer patients engage with these substances compared to alcohol. These findings reveal significant variability in substance use and perceptions among patients with psychotic disorders, highlighting the need for further investigation into the factors influencing these behaviors to develop effective treatment strategies and support systems tailored to this population.

**Disclosure of Interest:**

None Declared

